# Targeting Post-Translational Modifications to Improve Combinatorial Therapies in Breast Cancer: The Role of Fucosylation

**DOI:** 10.3390/cells12060840

**Published:** 2023-03-08

**Authors:** Gabriele Antonarelli, Valentina Pieri, Francesca Maria Porta, Nicola Fusco, Gaetano Finocchiaro, Giuseppe Curigliano, Carmen Criscitiello

**Affiliations:** 1 Division of New Drugs and Early Drug Development for Innovative Therapies, European Institute of Oncology, IRCCS, 20139 Milan, Italy; 2 Department of Oncology and Hemato-Oncology (DIPO), University of Milan, 20122 Milan, Italy; 3 Neural Stem Cell Biology Unit, Division of Neuroscience, IRCCS San Raffaele Hospital, 20132 Milan, Italy; 4 Vita-Salute San Raffaele University, 20132 Milan, Italy; 5 Division of Pathology, European Institute of Oncology (IEO), IRCCS, 20141 Milan, Italy; 6 School of Pathology, University of Milan, 20122 Milan, Italy; 7 Neurology Unit, IRCCS San Raffaele Scientific Institute, 20132 Milan, Italy

**Keywords:** fucosylation, glycosylation, breast cancer, metastasis, biomarkers

## Abstract

Various tumors rely on post-translational modifications (PTMs) to promote invasiveness and angiogenesis and to reprogram cellular energetics to abate anti-cancer immunity. Among PTMs, fucosylation is a particular type of glycosylation that has been linked to different aspects of immune and hormonal physiological functions as well as hijacked by many types of tumors. Multiple tumors, including breast cancer, have been linked to dismal prognoses and increased metastatic potential due to fucosylation of the glycan core, namely core-fucosylation. Pre-clinical studies have examined the molecular mechanisms regulating core-fucosylation in breast cancer models, its negative prognostic value across multiple disease stages, and the activity of in vivo pharmacological inhibition, instructing combinatorial therapies and translation into clinical practice. Throughout this review, we describe the role of fucosylation in solid tumors, with a particular focus on breast cancer, as well as physiologic conditions on the immune system and hormones, providing a view into its potential as a biomarker for predicating or predicting cancer outcomes, as well as a potential clinical actionability as a biomarker.

## 1. Introduction

Breast cancer is the most common malignant tumor worldwide, accounting for 31% of female cancers [1]. While early-stage breast cancer is characterized by a 5-year survival rate of 96% in Europe, metastatic disease is still incurable, with a 5-year survival rate of 38%. Novel treatment options are currently challenging this paradigm in metastatic breast cancer, mainly by targeting specific molecular alterations or metabolic vulnerabilities [2,3]. Moreover, breast cancer is a heterogeneous disease that can be divided into three broad groups: hormone receptor (HR) positive tumors based on estrogen and/or progesterone receptor (ER, PgR) status; human epidermal growth factor receptor 2 (HER2) positive tumors; or triple-negative breast cancers (TNBC). In addition, tumor heterogeneity also derives from multiple interactions between tumor cells and hosts’ related factors, such as the immune system or the hormonal axis. Overall, these factors are currently being exploited to improve the specificity of breast cancer diagnosis, prognosis, and therapeutics [4].

In this scenario, cancer glycosylation has been recognized as a key player related to tumor metabolism, aggressive clinical behavior as well as therapy resistance, with proposed roles as a predictive and prognostic biomarker [5]. Fucosylation is a specific type of glycosylation characterized by the transfer of a fucose residue from Guanosine Diphosphate (GDP)-fucose to oligosaccharide chains [6]. Cancer fucosylation, in particular within the glycan core, is linked to cellular aggressiveness, proliferation, and metastatic seeding across different pathologies, and its pre-clinical inhibition has been shown to delay tumor growth as well as to synergize with various anti-cancer therapeutics [7].

In the present work, we discuss the role of fucosylation in cancer, with a specific focus on breast cancer and its interactions with the immune and hormonal systems, providing an outlook on its applications as a biomarker as well as a novel therapeutic vulnerability.

## 2. Fucosylation: General Principles & Regulation

### 2.1. Fucose-Synthesis Pathways

**Figure 1:**
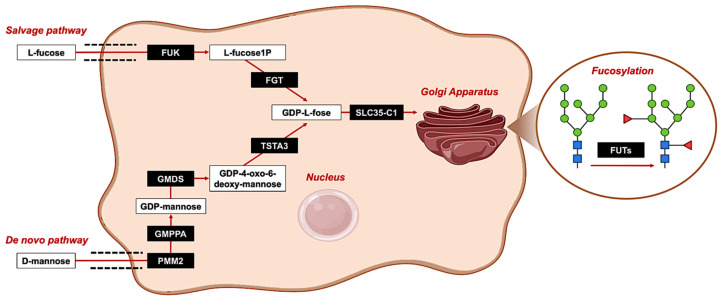
Fucose biosynthetic cellular pathways. The representative figure depicts fucose biosynthetic pathways, namely the *salvage pathway* (**top**) and the *de novo pathway* (**bottom**). On the one hand, 90% of GDP-L-fucose biosynthesis derives from the *de novo pathway*: D-mannose is processed by GDP-mannose-phosphorylase A (GMPPA), GDP-mannose 4,6-dehydratase (GMDS) and tissue-specific transplantation antigen p35B (TSTA3). On the other hand, 10% of GDP-L-fucose biosynthesis derives from the *salvage pathway*, in which free fucose derived from dietary intake is recycled by fucose kinase (FUK) and fucose-1-phosphate guanylyltransferase (FPGT). Then, the newly synthesized GDP-L-fucose is carried from the cytoplasm to the Golgi apparatus by the specific transporter SLC35C1. It is finally within the Golgi that GDP-L-fucose is conjugated to glycopeptides by highly specialized enzymes called FUTs. Abbreviations: PMM2: phosphomannomutase 2; GMPPA: GDP-mannose-phosphorylase A; GMDS: GDP-mannose 4,6-dehydratase; TSTA3: tissue specific transplantation antigen p35B; FUK: fucose kinase; FPGT: fucose-1-phosphate guanylyltransferase; SLC35C1: Solute Carrier Family 35 Member C1; FUTs: fucosyltransferase enzymes.

Fucose (6-deoxy-L-galactose) is the only levorotatory sugar synthesized and utilized by mammals and plays a fundamental role in the process of oligosaccharides post-translational modification. It can be integrated into the terminal portions of N-, O- or lipid-linked oligosaccharide chains through terminal-fucosylation, it can rearrange the core of complex N-glycans via core-fucosylation, or it can be straightly attached to threonine or serine residues in some glycoproteins [8]. All these processes are orchestrated by fucose synthesis, fucose transport from the cytoplasm to the Golgi apparatus, and, once there, fucose-residues transfer on glycan chains (Figure 1). The former happens via the *de novo pathway* for 90% of GDP-fucose biosynthesis when D-mannose is modified by three enzymes: GDP-mannose-phosphorylase A (GMPPA), GDP-mannose 4,6-dehydratase (GMDS) and tissue-specific transplantation antigen p35B (TSTA3). The remaining 10% of GDP-fucose synthesis relies instead on the *salvage pathway*, in which fucose kinase (FUK) and fucose-1-phosphate guanylyltransferase (FPGT) exploit free fucose derived from dietary intake [9]. The only GDP-fucose transporter identified so far is SLC35C1, which was found to be upregulated in hepatocellular and colorectal carcinoma [5]. GDP-fucose attachment to glycopeptides is mediated by thirteen fucosyltransferase enzymes (FUTs), categorized into five groups depending on the type of linkage, of which only FUT8 (a1-6 fucosyltransferase) catalyzes the core-fucosylation of the innermost N-Acetylglucosamine (GlcNAc) residue of N-glycans at the C6 position (Figure 1) [7,8,9].

### 2.2. Fucosylation in Various Cancers

Core-fucosylation is the most common form of fucosylation and has been associated with inflammation and cancer aggressiveness [9]. In particular, core-fucosylation has been related to inferior prognosis, as well as to increased proliferation, metastatic potential, and therapy resistance in melanoma [10], hepatocellular carcinoma [11], lung cancer [12,13,14], prostate cancer [15,16], pancreatic ductal adenocarcinoma [17], glioblastoma [18] and breast cancer [19,20].

The connection between aberrant core-fucosylation and melanoma dissemination was proven both in vitro and in vivo, firstly associating FUT8 overexpression with metastatic tumors by glycomics and then ‘wet’ validating its role in regulating cell invasion and migration. L1CAM was identified as one of the main proteins that, once core-fucosylated, mediates a pro-invasive phenotype in melanoma [10]. Another important process shown to be related to FUT8 up-regulation is the epithelial-to-mesenchymal transition (EMT), studied in the context of non-small cell lung cancer (NSCLC). Besides being significantly correlated with tumor recurrences and metastases, the FUT8 increase seemed to be triggered by β-catenin/lymphoid enhancer-binding factor-1 (LEF-1) signaling [14]. The EMT corresponding process in glioblastoma is referred to as proneural-to-mesenchymal transition (PMT), meaning that cancer cells in the neural/oligodendrocytes-progenitor-like state tend to adapt to hypoxic environments and become chemo-radioresistant by shifting towards a more malignant mesenchymal-like state. According to the findings on EMT in NSCLC, also PMT in GBM has been recently associated with the increase in FUT8 expression and core-fucosylation, in parallel with significantly faster tumor growth and matrix invasion. When tested on patient-derived tissues, FUT8 protein, and core-fucosylation resulted mostly upregulated in the restricted subset of mesenchymal-like GBM, and it was associated with dismal prognosis [18].

The reliable prognostic value of specific core-fucosylated antigens used as new cancer biomarkers has already been demonstrated in recent works. Indeed, the increase in core-fucosylated alpha-fetoprotein (AFP) in the serum of HCC patients can indicate cancer progression more specifically than the increase in total AFP [21]; in addition, the role of core-fucosylated haptoglobin has been described in the diagnosis of pancreatic cancer [22]. Although over the last few years, several works have demonstrated that core-fucosylation modulates numerous oncobiological events, it is important to emphasize that also aberrant O- and N-linked glycan structures expressed by transformed cells are able to influence the progression of different cancers and have been pointed as potential therapeutic targets [23,24,25,26,27].

### 2.3. Focus on Fucosylation in Breast Cancer

Several studies have investigated the role of the terminal- and core-fucosylation in clinical samples obtained from breast cancer patients utilizing different methodologies.

At first, liquid chromatography and mass spectrometry (LC-MS) were the methods of choice to study patterns of glycosylation. However, these techniques are not able to preserve tissue histology [28]. Building on this, the matrix-assisted laser desorption ionization MS imaging (MALDI-MSI) was introduced to allow for direct local N-glycans detection from tissue surfaces while preserving the histopathological architecture [29].

The first application of MALDI-MSI in breast cancer clinical samples involved the analysis of primary tumors. Indeed, breast cancer regions were characterized by a series of fucosylated, high-mannose, branched glycans with diverse specific N-glycans distribution between HER2+ and TNBC samples [30]. Moreover, changes in glycosylation patterns were also detected in necrotic tissues, which lack fucose modifications and display limited branching as well as sialic acid modifications [31]. By combining MALDI-MSI with hydrophilic interaction ultra-high performance liquid chromatography, Herrera et al. also identified a negative prognostic value of a specific core-fucosylated tetra-antennary N-glycan (F(6)A4G4Lac1), also associated with lymph node metastasis and disease recurrence, in breast cancer patients [32]. Stemming from these works, Ščupáková et al. utilized MALDI-MSI to study glycosylation variations between primary to metastatic lesions from 17 patients with advanced breast cancer from a rapid autopsy program. Of note, the authors found a progressive increase in N-glycan from normal breast tissues to primary tumors up to metastatic lesions, suggesting the potential future diagnostic and therapeutic unmet potential of high-mannose, fucosylated, and complex N-glycans in the advanced clinical setting. In particular, bone metastases displayed the most pronounced increase in core-fucosylation, mirrored by a decrease in high-mannose glycans [33].

Another recognized role of fucosylation in breast cancer has been shown in tumor angiogenesis and vascularization. In these regards, terminal-fucosylation of the clusterin glycoprotein is a cancer-specific post-translational modification found mainly in human luminal breast cancer. This alteration allows for the interaction between a fucosylated cluster and a C-type lectin (DC-SIGN), found on macrophages/myeloid cells, promoting the production of pro-angiogenic cytokines (i.e., vascular endothelial growth factor, VEGF; IL-8) while hampering the expression of HLA-DR [34].

Concerning the role of core-fucosylation as a biomarker in breast cancer, immunohistochemical and tissue microarray analyses of *FUT8* have demonstrated an association between high *FUT8* levels, lymph node metastases, and disease stage, also retaining a negative prognostic value by associating to reduced disease-free and overall survival [35]. However, to date, no core-fucosylated biomarker has been validated for breast, neither for predictive nor for prognostic purposes [36]. Glycoproteomic analyses from tumor tissue as well as from plasma of breast cancer patients may inform novel biomarkers and provide distinctive therapeutic vulnerabilities.

### 2.4. Regulation of FUT8 and Core-Fucosylation

Since FUT8 is essential in regulating core-fucosylation, the precise assessment of its regulation at the cellular level is of utmost importance. Epigenetic analyses have revealed low levels of *FUT8* methylation in cancers such as hepatocellular carcinoma, suggesting that the most relevant part of *FUT8* regulation may be at the transcriptional and post-transcriptional levels [37], a largely unexplored area of investigation.

#### 2.4.1. Transcriptional Regulation

The key players of *FUT8* transcription have not been completely elucidated yet. Genomic analyses of the *FUT8* gene, encoded on chromosome 14q23.3, have revealed the presence of at least nine exons, with eight exons spanning the coding sequence, as well as the presence of at least three different promoters [38]. More in detail, exon 1 encodes only for 5′ Untranslated Regions (UTR) sequences containing potential binding sites for transcription factors (i.e., TATA-box, cMyb, GATA-1, bHLH) [39]. Of note, a positive transcriptional axis has been recognized in melanoma with Transforming Growth Factor-β (TGF-β)-Induced Factor homeobox 2 (TGIF2) [10], whereas a negative one has been depicted with the transcription factor ASCL1 in small cell lung cancer [40] and glioblastoma [18]. Information on the relevance of TGIF2 and ASCL1 in *FUT8* regulation in breast cancer is still lacking [10,40].

**Figure 2:**
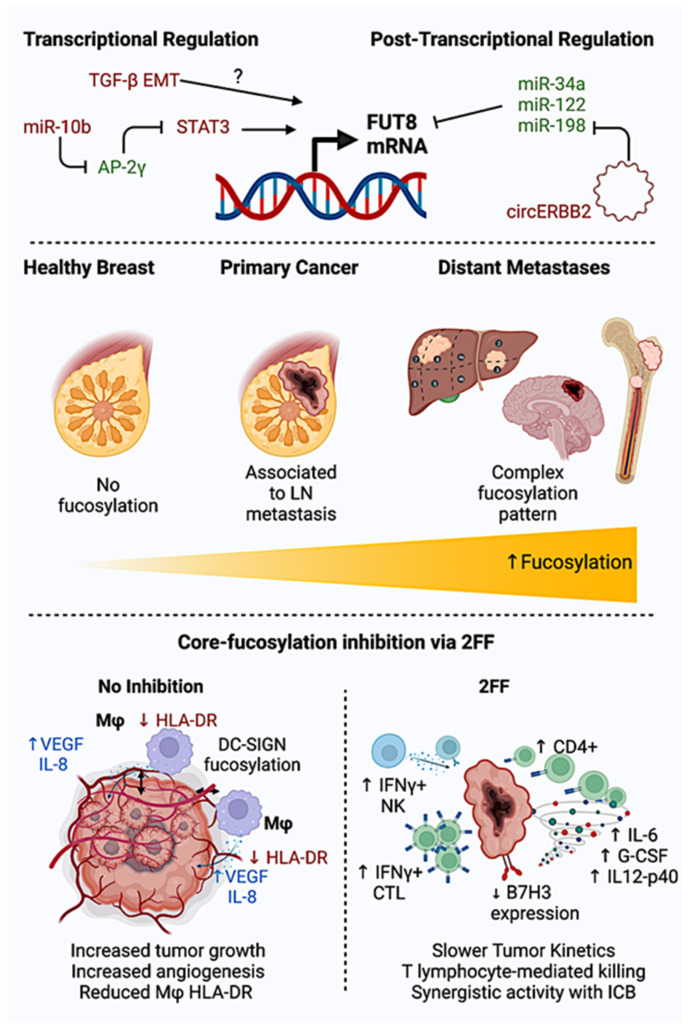
Fucosylation in Breast Cancer (BC): Biomarker and Therapeutic Vulnerability. Role of Fucosylation in BC. Top: Transcriptional and post-transcriptional regulation of *FUT8* expression in BC (red: promoting *FUT8* transcription; green: inhibiting *FUT8* transcription/translation). Middle: Fucosylation as a biomarker across disease stages, showing no expression in healthy mammary glands, intermediate expression in locally advanced disease (associated with lymph node metastasis), and highest expression in metastatic disease. Bottom: Pharmacological inhibition in pre-clinical tumor models via 2FF induces CTL-/NK-mediated tumor killing, and synergistic activity with immune checkpoint inhibitors (i.e., anti-PDL1 mAb), ultimately reducing tumor growth kinetics. Abbreviations: LN: lymph node; TGFβ: Transforming Growth Factor beta; miR: microRNA; circERBB2: circular *ERBB2* RNA; EMT: epithelial to mesenchymal transition; AP-2γ: activator protein 2γ; STAT3: Signal Transducer and Activator of Transcription 3; FUT8: fucosyltransferase 8; PDL1, Programmed Death Ligand 1; mAb, monoclonal antibody; Mϕ: macrophage; VEGF: vascular endothelial growth factor; CTL: cytotoxic T-lymphocytes; ICB: immune checkpoint blockade; HLA-DR: Human-leukocyte-associated Antigen-DR; IL: interleukin; IFNγ: interferon-gamma; 2FF: 2-fluorofucose; G-CSF: granulocyte colony-stimulating factor; DC-SIGN: Dendritic Cell-Specific Intercellular adhesion molecule-3-Grabbing Non-integrin. Created with BioRender.com.

One proposed mechanism of *FUT8* induction in breast cancer has been recognized upon TGFβ-induced EMT (Figure 2). While FUT8 overexpression acts as a stimulus to TGF-β-induced EMT, *FUT8* knockdown suppresses cell invasiveness and metastatic potential. However, the exact molecular players driving this axis have not yet been identified, with β-catenin/lymphoid enhancer-binding factor-1 or E-box-binding transcription factors (i.e., SNAIL or TWIST) being the first candidates due to structural promoter sequences and to data obtained in other tumor settings [14,20].

Another work has unveiled a *FUT8* regulatory axis in breast cancer based on the transcription factor activator protein 2γ (AP-2γ) binding to the Signal Transducer and Activator of Transcription 3 (STAT3) [41]. This complex prevents STAT3 phosphorylation and STAT3-mediated *FUT8* transcription. Co-immunoprecipitation assays have shown strong interactions between AP-2γ and STAT3 (but not phospho-STAT3), and chromatin immunoprecipitation analysis has revealed phospho-STAT3 binding to *FUT8* promoter (Figure 2) [41]. Of note, apart from promoting an immune-suppressive tumor microenvironment, STAT3 signaling in breast cancer cells not only contributes to proliferation and metastatic behavior but also mediates immune evasion and resistance to cyclin-dependent kinase inhibitors (CDKi) [42,43]. For the abovementioned reasons, STAT3 is considered a therapeutic target in breast cancer, although direct targeting has shown major hurdles in pharmacokinetic profiles, with indirect or combinatorial targeting currently entering clinical testing [44,45,46].

#### 2.4.2. Post-Transcriptional Regulation: miRNAs

In vitro studies utilizing hepatocellular carcinoma cell lines demonstrated via a luciferase reporter technology that two microRNA, miR-34a and miR-122, play a negative role in *FUT8* post-transcriptional regulation by interacting with the *FUT8* 3′-UTR and they ultimately can modulate glycosylation patterns [47]. Interestingly, in a cohort of 25 breast cancer patients, both miR-34a and miR-122 have been detected as circulating microRNAs upon neo-adjuvant chemotherapy. Their levels were significantly upregulated in patients with breast cancer, achieving a complete pathological response (pCR) after neoadjuvant chemotherapy [48]. Specific cellular contributors to miR-34a and miR-122 expression need to be fully elucidated and targeted (Figure 2).

Another miRNA characterized as a possible *FUT8* regulator is miR-10b, with a proposed positive impact on core-fucosylation, cellular motility, and proliferation [19]. Mechanistically, miR-10b has been shown to downregulate the transcription factor AP-2γ, which in turn binds to STAT3, preventing its phosphorylation, hence *FUT8* transcription [41]. miR-10 b’s role in metastatic breast cancer patients has been suggested to be crucial in advanced disease based on the activation of the Twist transcription factor [49]. However, conflicting data have been collected by evaluating the prognostic role of miR-10b in breast cancer patients [50,51]. Overall, glycosylation data and proteomic analysis clearly depicting the influence of miR-10b on *FUT8* and on cellular fucosylation patterns are still lacking and need to be assessed thoroughly.

miR-198 also acts as a direct negative regulator of *FUT8* expression both in a colorectal and a non-small cell lung cancer model, with miR-198 inhibition leading to an aggressive phenotype and a survival disadvantage [52,53]. While the mechanistic link between miR-198 and *FUT8* has not yet been investigated, it has been shown that circular ERBB2 RNA (circ-ERBB2) promotes breast cancer metastatic process, cellular invasion, and proliferation by competing with miR-198 and miR-136-5p as an endogenous RNA sponge (Figure 2) [54,55].

## 3. Fucosylation as Druggable Target: From Pre-Clinical Studies to Clinical Translation

### 3.1. Specific Fucosylation Inhibitors

RNAi silencing of *FUT8* has been shown to reduce core-fucosylation of cancer cells and functionally inhibit their migration and invasion in vitro [10,18], as well as tumor growth capacity in vivo [18]. To provide patients with feasible treatment protocols, genetic engineering strategies cannot be directly translated into clinics, and a handy drug is needed. It is currently the most promising strategy to reduce fucosylation in cancer to develop orally acting fucose analogs that compete with physiological fucose in the Golgi and engulf the fucosyltransferase machinery [56]. In this context, 2-Fluoro-Fucose (2FF), a cell-permeable fluorinated fucose derivative, has been tested in both pre-clinical models as well as in human patients as a treatment for a variety of cancer types after oral, intraperitoneal (IP), or intravenous (IV) administration (Table 1).

### 3.2. 2FF Use in Various Cancers

To our knowledge, Okeley et al. were the first to test 2FF in vivo and demonstrated the efficacy of different compounds in enhancing ADCC activity of monoclonal antibodies (mABs) and inducing reversible neutrophilia and also demonstrated that the drug had a direct anti-tumor effect in lymphoma and colorectal cancer models [58]. They obtained systematic data on tolerability and bioavailability for oral (drinking water or gavage), IP, and IV administration schedules, paving the way for future studies [58]. 2FF was evaluated in the context of hepatocellular carcinoma, where increased levels of core-fucosylation are already associated with worse outcomes [11]. As a result of demonstrating significant inhibition of HepG2 cell proliferation and integrin-mediated cell migration in vitro, Zhou et al. found that, after inoculating HepG2 cells pre-treated with 2FF and then injected with intra-tumoral drug injections, the tumor volume shrank consistently in subcutaneous HCC models [11].

In line with those findings, Pieri et al. examined the role of core-fucosylation in the mesenchymal subgroup of glioblastoma (GBM), the one associated with worse prognosis and chemoradiation resistance [18]. In orthotopic xenografts of human GBM, 2FF delivered intratumorally via micro-infusion pumps resulted in significantly reduced tumor volume and increased survival. Moreover, glycoproteomic profiling of patient-derived GBM cells revealed high levels of core-fucosylated proteins related to extracellular matrix adhesion and integrin-mediated signaling pathways, fundamental mediators of tumor aggressiveness, which are turned off by 2FF treatment [18].

Aside from testing 2FF as a monotherapy, combinatorial approaches with immunotherapies also appeared promising. Based on the finding that core-fucosylation is required for proper PD1 expression and ligand-receptor interaction, Okada et al. tuned the post-translational regulatory mechanisms of PD1 in order to optimize the anti-tumor immune response [61]. In particular, 2FF attenuated PD1 expression in T cells and strengthened their antitumoral attack against melanoma, further supporting its use in combination with pembrolizumab [61].

### 3.3. Focus on 2FF Use in Breast Cancer

Two genetically distinct transgenic breast cancer models have been shown to benefit from therapeutic fucosylation inhibition via 2FF-the TgMMTV-neu (HER2+ luminal B) and the C3(1)-Tag (basal-like) [63]. Compared to those isolated from untreated mice, IgG isolated from treated mice showed enhanced tumor cell lysis, suggesting enhanced ADCC function and tumor-specific reactogenicity. Moreover, 2FF treatment at two different doses in a prophylactic anti-tumor experimental setting delayed tumor formation, prevented cancer development in 33% of TgMMTV-neu and 26% of C3(1)-Tag models, and enhanced splenocyte reactogenicity upon exposure to tumor-lysate. Additionally, pro-inflammatory cytokines (such as interleukin-6, IL-6; IL12-p40; and granulocyte-colony stimulating factor, G-CSF) were elevated throughout the body. Importantly, the anti-tumor effect of 2FF was greatly reduced upon CD4 T cell depletion, suggesting an active role of the immune system in mediating the anti-cancer activity upon fucosylation inhibition [63].

The role of fucosylation in modulating anti-cancer immunity and combining therapeutic approaches has also been characterized in a TNBC pre-clinical model, primarily using 4T1 cells [65]. In this work, Huang et al. first confirmed that excessive glycosylation of the immune-suppressive checkpoint B7-H3 protein, present on tumor and/or antigen-presenting cells, retains a negative prognostic value in TNBC patients. The N-glycosylation of B7-H3 at Asn-X-Ser/Thr motifs (where X is any amino acid except proline) leads to increased stabilization and membrane expression. The key enzyme involved in this glycosylation step was shown to be FUT8, which positively correlated with *B7-H3* mRNA expression, but not transcription and also correlated with worse prognosis in patients with TNBC. As a result of scoring FUT8 immunohistochemical (IHC) expression by membrane intensity and percentage of positive cells, patients were almost equally divided into low- and high-groups, indicating that FUT8 expression in TNBC patients is supposedly heterogeneous. Functionally, B7-H3 core-fucosylation led to reduced immune system engagement, as evidenced by in vitro and in vivo experiments. Both B7-H3 wild-type and B7-H3-4NQ tumors grew similarly in SCID mice, but the former showed faster kinetics in syngeneic, immunocompetent BALB/c mice. B7-H3 wild-type tumors also had a reduced infiltration of T lymphocytes, both CTLs and CD4, as well as NK cells. To further corroborate these findings, treatment of B7-H3 wild-type tumors in syngeneic mice with both the core-fucosylation inhibitor 2FF and anti-PDL1 mAb resulted in reduced tumor growth kinetics, decreased B7-H3 expression on tumor cells, and in increased infiltration of IFNγ+ NK cells as well as of IFNγ+ CD8 or CD4 T lymphocytes [65].

Overall, these studies provide evidence that core-fucosylation plays a significant role in tumor biology, invasiveness, metastatic seeding, as well as tumor-immune interactions. The research supports the use of fucosylation inhibitors, including 2FF, in various clinical situations, including breast cancer, either alone or in combination with immune-stimulating therapies.

### 3.4. First 2FF-Based Clinical Trial

The abovementioned pre-clinical data prompted for clinical testing of 2FF in a First-in-Human, First-in-Class, Phase 1 clinical trial in patients with advanced solid tumors, either alone or in combination with pembrolizumab (NCT02952989) [66]. A total of 46 patients were enrolled, mostly (33/46) in part A dose-escalation monotherapy arm. A dose-proportional pharmacokinetic profile, with target inhibition of fucosylation, was demonstrated, with the identification of the Maximum Tolerated Dose (MTD) of 10 g daily. According to RECIST v1.1 criteria, 10 patients (36%) reached stable disease after 10 cycles among the 28 patients evaluated for response in part A, of whom one patient with triple-negative breast cancer showed a 51% disease reduction and a partial response (PR) based on immune-related RECIST criteria. While nausea, fatigue, and diarrhea were the most common toxicities (47%) in part A and part C, thromboembolic events (grades 2–5) were detected in 16% (5/32) and 14% (1/7) of patients, despite concurrent prophylactic anticoagulation, which led to the early termination of the study [66]. As a result of this experience, the oral 2FF fucosylation inhibitor was found to have promising anti-tumor activity, which may be exploitable as monotherapy or in combination with other therapies in the clinic in the future. However, a more refined selection of patients, alternative thromboembolic drug prophylaxis, and/or second-generation inhibitors are clearly required.

## 4. Fucosylation Interplay with the Immune System and Hormonal Pathways

### 4.1. Macrophages

Macrophages play key roles in multiple immunological and cancer-related processes, such as antigen uptake and presentation, angiogenesis, metastatic seeding, and chemotherapy resistance [67,68]. They modulate the microenvironment by integrating multiple signals, and their modulation ranges from the inflammatory, M1-like, to the immune-modulatory, M2-like polarization [69].

Rheumatoid arthritis (RA) patients’ synovial cells express terminal-fucosylation but not core-fucosylation, and this expression correlates positively with tumor necrosis factor-alpha (TNFα). In vitro, terminal-fucosylation inhibition by 2-deoxy-D-galactose (2-D-gal), blocking FUT1/2 enzymes, resulted in suppression of M1 differentiation and in M1 to M2 polarization. In vivo, 2-D-gal dramatically reduced the onset of collagen II-induced arthritis [70]. *Fut8* KO macrophages also showed altered CD14 and Toll-like Receptor (TLR) 2 and 4 axis expression in an experimental model of lipopolysaccharide (LPS) stimulation. As a result, mice transplanted with *Fut8* KO hematopoietic bone marrow displayed enhanced resistance to inflammation [71]. In addition, macrophages represent one the largest leukocyte population in various tumor microenvironments and have been described to display unusual glycosylation patterns, which have, in turn, been proposed as potential therapeutic targets [72,73,74,75].

### 4.2. T & B Lymphocytes

Glycosylation is a recognized modulator of lymphocytes’ functions, from autoimmunity to cell activation and homeostasis [76]. O-fucosylation regulates T cell development as well as lymphoid/myeloid fate specification in hematopoietic progenitors through Notch signaling [77]. *Fx* KO mice show an expansion of myelopoiesis and a contraction of lymphopoiesis [78]. The O-fucosylation of Notch1/2 by Protein O-Fucosyltransferase 1 (POFUT1) promotes B-cell and thymocyte development while reversing the myeloproliferative burst in Pofut1 knockout mice [79].

Moreover, ex vivo fucosylation of Cytotoxic T Lymphocytes (CTLs), while not affecting target specificity, has been shown to enhance homing and tumor cell killing [80]. Interestingly, while broad fucosylation inhibition via 2-FluoroFucose (2FF) has been proposed to positively impact T Cell Receptor (TCR) engagement and regulation [81], core-fucosylation of the heavy chain of the B Cell Receptor (BCR) is needed for proper B cell development and transition from the pro-B stage. Moreover, *Fut8* KO mice display reduced immunoglobulin (Ig) production (IgG, IgA, IgM) [82,83]. In addition, IgG production is improved upon fucose recognition by DC-SIGN on dendritic cells (DCs), facilitating T follicular helper (T_FH_) cells’ differentiation [84].

Lastly, core-fucosylation influences immunologically relevant co-receptors, thereby influencing the cancer immunity cycle. The programmed death 1 (PD-1) receptor is indeed regulated by post-translational modifications, such as core-fucosylation, and blockade of it in a pre-clinical model of melanoma using 2FF has been shown to enhance T cell-driven antitumoral immunity by reducing PD-1 membrane expression [61].

### 4.3. Antibodies

Even though antibodies exhibit highly conserved structures, with variable heavy and light chains conferring specificity, post-translational modifications have been shown to significantly impact their effector functions, with fucosylation being the most studied [85,86,87]. As a matter of fact, afucosylated Fc glycans exhibit a high affinity for FcgRIIIa glycans, increasing antibody-dependent cell cytotoxicity (ADCC) [88,89], and this property is already being exploited in drug engineering, as with Amivantamab, a bispecific antibody [90,91]. In vivo, 2FF exposure has been linked with afucosylated IgG production and displayed an anti-tumor effect in both syngeneic and xenograft models [58]. Individuals from different geographical regions have different Ig glycosylation profiles [92]; in contrast, viral-vectored vaccines can produce similar antigen-specific IgG glycosylation profiles that are influenced by inflammatory stimuli after B cell priming [93].

There is also evidence indicating that Ig fucosylation plays a role in infectious diseases, with reduced levels found in HIV elite controllers [94] and a link between dengue and COVID-19 severity [95,96]. Interestingly, afucosylated anti-SARS-CoV2 Ig increased inflammation by activating macrophages and produced prothrombotic conditions [97,98].

### 4.4. Hormonal Pathways

Both men and women have shown that estrogen regulates IgG glycomic composition. Indeed, post-menopausal women display increased pro-inflammatory IgG glycoforms lacking terminal galactose. Such agalactosylated IgGs display enhanced complement fixation via lectin pathways and ADCC. Interestingly, testosterone aromatization has also been shown to cause such estradiol-related events in men [99].

Furthermore, it was found that perimenopause is associated with decreased galactosylated glycans and increased IgG core-fucosylation by studying IgG glycome changes. In addition to promoting low-grade inflammation due to a loss of galactosylation, increased core-fucosylation has also been linked to less efficient Ig effectors [100].

Overall, these data show the vast interplay between the immune/hormonal systems and protein fucosylation, highlighting a relevant role of such post-translational modifications on relevant physiologic processes in healthy and diseased conditions and suggesting possible novel biomarkers to be investigated as well as therapeutic vulnerabilities to be addressed.

## 5. Discussion

Solid tumors utilize multiple complex mechanisms to facilitate cellular growth, adapt to hostile environments, evade immune recognition, and develop resistance to various therapeutic approaches. Many of these characteristics are shared among different malignancies and have been thoroughly characterized, ultimately leading to the identification of novel, tailored therapeutic approaches [101]. Solid tumors often develop a hypoxic, largely immune-suppressive tumor microenvironment (TME), which ultimately poses an insurmountable obstacle to anti-cancer therapies, including cell therapies [102,103]. In addition, malignant tumors have been shown to hijack post-translational modifications, such as glycosylation and fucosylation, to block cell-to-cell communications [104,105].

There is a growing body of evidence that fucosylation plays a role in regulating the immune and hormonal systems under physiological conditions, but many questions remain, especially when it comes to large, prospective population studies. In addition, fucosylation has also been convincingly identified as a cancer-related characteristic enabling tumor invasiveness, aggressiveness, angiogenesis, and immune evasion in several solid malignancies [6]. Clinical samples from breast cancer patients have also revealed exaggerated core-fucose PTMs upon disease progression and metastatic spread. Further, pharmacologic inhibition of fucosylation in different breast cancer pre-clinical models has shown significant anti-tumor activity, also linked to immune responses [10,18,56,58]. In line with this, synergism between fucosylation inhibitor 2FF with the anti-PD1 immune checkpoint blockers has also been documented, instructing for hypothetical combinatorial treatment strategies [61].

To date, fucosylated proteins have been largely unexplored as a prognostic or predictive biomarker. While in patients with hepatocellular carcinoma, the level of fucosylated, rather than total, alpha-fetoprotein was shown to be more specifically associated with cancer progression [21], no such specific biomarker currently exists for breast cancer. Considering this, the study of peculiar fucosylated biomarkers would be of great clinical value, especially in areas of unmet clinical need, such as in adjuvant clinical decision-making.

It has been suggested that fucosylation and, in particular, core-fucosylation is a newer hallmark of cancer that can influence cell-to-cell communications, foster the development of derailed TMEs, and ultimately influence resistance to chemotherapy. In spite of the fact that most of the molecular regulators of cancer-related core-fucosylation are still largely unknown, as well as most of their functional implications, more sustained pre-clinical research is urgently needed in the coming years to guide further refined clinical testing in the future.

## Figures and Tables

**Table 1:** Pre-clinical and clinical studies evaluating the effects of anti-fucosylation drugs. Only one clinical trial has been published, while the other studies were performed on cells and murine models of various cancers and sickle-cell disease.

Reference	Pathology	Type of Anti-Fucosylation Drug	Treatment Platform	Dose	Treatment Effect
Rillahan et al., 2012 [57]	Human HL-60 promyelocytic leukemia cells, CHO cells	2FF (2-Fluoro-Fucose, SeaGen) 6FF (6-Fluoro-Fucose, SeaGen)	In vitro: Drug in cell medium	From 2 μM to 512 μM (screening of different drugs at many dosages. Drop in fucosylation observed starting from 32 μM)	Almost complete abolition of Lewis X and SLe^X^ epitopes. Inhibition of overall fucosylation
Okeley et al., 2013 (*) [58]	LS174T colorectal cells, CHO cells	2FF 5-alkynylfucose 2FF and 5AF peracetylated derivatives (SeaGen)	In vitro: Drug in cell medium	From 50 μM to 1000 μM (screening of different drugs at many dosages. Full inhibition of mABs-fucosylation observed already at the lowest drug concentration tested)	Production of low-fucose monoclonal antibodies (Enhanced ADCC)
	BALB/c mice implanted with A20 murine lymphoma cells IV; Nude mice implanted SC with LS174T colorectal cells		In vivo: Compounds injected IP and IV, or provided in drinking water/gavage	IP: 150 mg/kg daily for 1 week. Concentration in water: 100 mM	Reduced tumor volume and increased mice survival
Belcher et al., 2015 [59]	Transgenic sickle cell disease mice	2FF (SeaGen)	In vivo: Compounds provided in drinking water or by gavage	Concentration in water: 100 mM for 7 days. Gavage: 150 mg/mL (0.01 mL/g, twice per day) 1 or 3 days	Reduced microvascular stasis, leukocytes rolling/adhesion, NFKB activation. Increased WBC count
Kizuka et al., 2017 [60]	HEK293 cell line + its glycosylation mutant HEK293S/GnT-I. Hepatoma cell lines (Hep3B, HepG2, FTO2B)	2FF (SeaGen) peracetylated 6-Alk fucose	In vitro: Drug in cell medium	50 µM	Inhibited overall fucosylation. Reduced hepatoma cell migration and invasion
Zhou et al., 2017 (*) [11]	Human hepatoma HepG2 and Hela cell lines	2FF (SeaGen)	In vitro: Drug in cell medium	From 0.5 to 500 μM (screening of different drugs at many dosages. Drop in fucosylation observed starting from 20 μM)	Inhibited cell proliferation, migration, and colony formation
	BALB/c-nu mice implanted with human hepatoma HepG2 cells SC		In vivo: Compounds directly injected into each tumor tissue for 7 days, then once a week for 3 weeks.	100 μM	Reduced tumor volume and weight
Okada et al., 2017 (*) [61]	OT-I Th1 cells, activated	2FF (SeaGen)	In vitro: Drug in cell medium	100 μM	Reduced PD-1 expression
	Mice implanted SC with B16-ovalbumin (OVA) melanoma cells		In vivo: OT-I Th1 cells pre-treated in vitro, then injected IV in mice or in monotherapy, or in combination with pembrolizumab	100 μM	In monotherapy, reduced OVA cells growth. In combination with pembrolizumab, more durable therapeutic response
McKenzie et al., 2018 [62]	Murine hybridoma cell lines	6,6,6-trifluoro-L-fucose (F3Fuc) (Derived from mannolactone by the authors)	In vitro: Drug in cell medium	10 mM	Production of low-fucose monoclonal antibodies (Enhanced ADCC)
Zimmermann et al., 2019 (*) [56]	CHO-K1 cell line producing a recombinant IgG1	5-alkynylfucose (Carbosynth), 5-alkynylfucose peracetate (Thermo Fisher scientific), 2-deoxy-2-fluorofucose (Cayman Chemical), 2F-peracetyl-fucose (Thermo Fisher scientific)	In vitro: Drug in cell medium	Concentrated stock solutions in DMSO, due to reported water insolubility: 34.2 mM PerAcFuc 150 mM 2F-Fuc 50 mM 5-AlkFuc 50 mM 5-AlkFucPerAc Then: screening of different drugs at many dosages, from 200 to 800 μM	Reduced IgG1 glycosylation (Dose-dependent effect)
Disis et al., 2020 (*) [63]	Transgenic breast cancer mice: TgMMTV-neu (luminal B) and C3(1)-Tag (basal)	2FF (SeaGen)	In vivo: drinking water	20 mM or 50 mM 2FF drinking water	Reduced tumor volume, delayed tumor onset, boosted anti-cancer immunity
Belur et al., 2020 [64]	Hepatoma cell line HepG2 and pancreatic cancer cell line PANC-1	Core-fucose specific lectins (CSL-*Cephalosporium curvulum;* AOL-*Aspergillus oryzae*; LCA-*Lens Culi-naris Agglutinin*	In vitro: Drug in cell medium	5 μg/mL	Increased cell apoptosis
Huang et al., 2021 (*) [65]	Human breast cancer cells MDA-MB-231	2FF (SeaGen)	In vitro: Drug in cell medium	300 μM	Repressed B7H3 expression, improved T cell activation
	Mice injected into mammary fat fad with breast cancer cells MDA-MB-231		In vivo: Tumor cells pre-treated in vitro, then mice received drug by gavage or in monotherapy, or in combination with pembrolizumab	Gavage: 3.51 mg/mL	Reduced tumor volume. In combination with pembrolizumab, further growth inhibition
Do et al., 2021 (*) [66]	BASKET TRIAL ON PATIENTS with refractory non-small cell lung cancer, squamous cell carcinoma of head&neck, CRC, breast, urothelial and renal cancer ClinicalTrials.gov NCT02952989	SGN-2FF (SeaGen)	In vivo (humans): SGN-2FF monotherapy administered orally or SGN-2FF administered orally in combination with pembrolizumab IV	1, 2, 5, 10, 15 g QD; 2 and 5 g b.i.d. grams (g) per flat dose	Dose-proportional pharmacokinetics, pharmacodynamic target inhibition of glycoprotein fucosylation, preliminary anti-tumor activity. Thromboembolic events led to study termination.
Pieri et al., 2022 (*) [18]	Patient-derived glioblastoma cell lines	2FF (SeaGen)	In vitro: Drug in cell medium	100–500 μM	Reduced cell fucosylation, self-renewal and proliferation
	Nude mice intracranially implanted with patient-derived glioblastoma cell lines		In vivo: Intratumoral drug delivery by mini-pumps, mimicking convection-enhanced delivery	6 µL/day of 2FF at 4 mM for 16 days or 3.6 µL/day of 2FF at 6.6 mM for 42 days	Reduced tumor growth, prolonged mice survival

(*): studies cited in the main text; CHO: Chinese hamster ovary cells; μM: micro-molar; mABs: monoclonal antibodies; ADCC: antibody-dependent cellular cytotoxicity; IV: intravenous; IP: intraperitoneal; NFKB: nuclear factor kappa-light-chain-enhancer of activated B cells; WBC: white blood cells; Th: T helper cells.

## Data Availability

No new data were created or analyzed in this study. Data sharing is not applicable to this article.
